# Polymeric nanoparticles for the delivery of siRNA and the partnership between WSU and UNIT

**DOI:** 10.1186/1753-6561-8-S4-O13

**Published:** 2014-10-01

**Authors:** Sandro da Rocha, Denise Conti

**Affiliations:** 1 Wayne State University, Detroit, MI, USA; 2 Universidade Tiradentes, Aracaju, Sergipe, Brazil; 3 Office of Generic Drugs, Science Group, Center for Drug Evaluation and Research, US Food and Drug Administration (FDA), Silver Spring, MD, USA

## 

RNA interference (RNAi) is an endogenous regulatory process whereby double-stranded RNA (dsRNA) present in the cell cytoplasm causes sequence-specific, post-transcriptional gene silencing [1]. dsRNA is first cleaved into short (19-21 nucleotides long) dsRNA fragments (siRNA) by Dicer, an endoribonuclease. The siRNA, which may be endogenous or exogenous (synthetically produced therapeutic) is subsequently loaded onto the RNA-induced silencing complex (RISC). The sense or passenger strand of the siRNA is then unwound and cleaved. The retained anti-sense or guide strand is highly specific to the target messenger RNA (mRNA) through complementary base paring. The activated RISC-siRNA complex scans, binds and degrades the complementary target mRNA present in the cell cytoplasm at a highly specific position relative to the 5' end of the anti-sense strand, thus causing degradation of the complementary mRNA, which then leads to gene silencing. This process is catalytic, allowing the same RISC-siRNA complex to cleave multiple copies of the target mRNAs, and will continue until the siRNA is degraded or diluted upon cell division.

With the decoding of the human genome siRNA has emerged as a broadly applicable and versatile therapeutic platform. The drug discovery aspect is greatly optimized for therapeutic siRNA compared to small molecular weight drugs, as one only requires knowledge of the sequence of the target gene in the case of siRNA, while trial and error through more or less random modification of the drug is required during screening of small drug molecules [2]. The specificity of siRNA is particularly relevant in the treatment of cancer, as there is the potential to selectively act only on cancerous cells, even when the therapeutic reaches non-target cell populations. siRNA therapy also holds promise in targeting the so-called 'non-druggable' diseases - those not amenable to conventional therapeutics such as small molecules, or monoclonal antibodies [3]. Another advantage of therapeutic siRNA is that gene regulation happens in the cell cytosol, and not the nucleus as in DNA-based gene therapies. This is significant, as the passive transport through the nuclear membrane is highly regulated, much more so than that across the cellular membrane, and is thus particularly relevant to postmitotic cells such as those in the airways.

The critical limiting factor in translating siRNA therapeutics to the clinic is the ability to efficiently deliver siRNA to the cell cytoplasm.[4]. Free siRNA is very unstable in the extracellular milieu, with plasma half-lives of only ca. 10 min [5] Moreover, due to its negative charge and large size, free siRNA is not readily internalized through passive diffusion, and is easily degraded in the lysosomes following cellular internalization through endocytosis [4,6]. Therefore, only a small fraction of the initial siRNA dose reaches the cell cytosol when administered as a free molecule. The use of nanocarriers for the efficient delivery of siRNA, and the ability to formulate such nanocarriers for regional delivery to the tissue of interest is thus of great relevance in developing siRNA therapeutic platforms.

Because viral carriers are potentially associated with severe immunological responses, non-viral siRNA carriers are preferred [6]. Non-viral (nano)carriers may involve complexation of siRNA with a positively charged carrier, chemical conjugation, or encapsulation [5,7]. The most widely used methods for the delivery of nucleic acids are lipoplexes, and polyplexes, and complexes/self-assembled domains. Conjugation of siRNA to singly functionalizable molecules such as PEG and cholesterol has been also explored. More recently, conjugation of siRNA to multivalent nanocarriers has been proposed, and this strategy may provide unique opportunities that may help overcome the challenges associated with the delivery of siRNA with complexes and singly functionalizable molecules. In this work we will discuss recent advances on the use of nanocarriers for siRNA delivery, with a special focus on the targeting of lung diseases. We will also discuss strategies for the formulation of such siRNA carriers for the regional delivery to the lungs using oral inhalation devices.

## References

[B1] Fire A Xu S Montgomery MK Kostas SA Driver SE Mello CC Potent and specific genetic interference by double-stranded RNA in Caenorhabditis elegans Nature 1998 391 806 11 10.1038/35888 9486653

[B2] Guo P Coban O Snead NM Trebley J Hoeprich S Guo S Engineering RNA for targeted siRNA delivery and medical application Adv Drug Deliv Rev 2010 62 650 66 10.1016/j.addr.2010.03.008 20230868 PMC2906696

[B3] Soutschek J Akinc A Bramlage B Charisse K Constien R Donoghue M Therapeutic silencing of an endogenous gene by systemic administration of modified siRNAs Nature 2004 432 173 8 10.1038/nature03121 15538359

[B4] Juliano R Alam MR Dixit V Kang H Mechanisms and strategies for effective delivery of antisense and siRNA oligonucleotides Nucleic Acids Res 2008 36 4158 71 10.1093/nar/gkn342 18558618 PMC2475625

[B5] Pecot CV Calin GA Coleman RL Lopez-Berestein G Sood AK RNA interference in the clinic: challenges and future directions Nat Rev Cancer 2011 11 59 67 10.1038/nrc2966 21160526 PMC3199132

[B6] Whitehead KA Langer R Anderson DG Knocking down barriers: advances in siRNA delivery Nat Rev Drug Discov 2009 8 129 38 10.1038/nrd2742 19180106 PMC7097568

[B7] Dominska M Dykxhoorn DM Breaking down the barriers: siRNA delivery and endosome escape J Cell Sci 2010 123 1183 9 10.1242/jcs.066399 20356929

